# *Agrobacterium* Mediated Transient Gene Silencing (AMTS) in *Stevia rebaudiana*: Insights into Steviol Glycoside Biosynthesis Pathway

**DOI:** 10.1371/journal.pone.0074731

**Published:** 2013-09-04

**Authors:** Praveen Guleria, Sudesh Kumar Yadav

**Affiliations:** CSIR–Institute of Himalayan Bioresource Technology, Palampur, Himachal Pradesh, India; National Taiwan University, Taiwan

## Abstract

**Background:**

Steviol glycoside biosynthesis pathway has emerged as bifurcation from ent-kaurenoic acid, substrate of methyl erythritol phosphate pathway that also leads to gibberellin biosynthesis. However, the genetic regulation of steviol glycoside biosynthesis has not been studied. So, in present study RNA interference (RNAi) based *Agrobacterium* mediated transient gene silencing (AMTS) approach was followed. *SrKA13H* and three *SrUGTs* (*SrUGT85C2*, *SrUGT74G1* and *SrUGT76G1*) genes encoding ent-kaurenoic acid-13 hydroxylase and three UDP glycosyltransferases of steviol glycoside biosynthesis pathway were silenced in *Stevia rebaudiana* to understand its molecular mechanism and association with gibberellins.

**Methodology/Principal Findings:**

RNAi mediated AMTS of *SrKA13H* and three *SrUGTs* has significantly reduced the expression of targeted endogenous genes as well as total steviol glycoside accumulation. While gibberellins (GA_3_) content was significantly enhanced on AMTS of *SrUGT85C2* and *SrKA13H*. Silencing of *SrKA13H* and *SrUGT85C2* was found to block the metabolite flux of steviol glycoside pathway and shifted it towards GA_3_ biosynthesis. Further, molecular docking of three SrUGT proteins has documented highest affinity of SrUGT76G1 for the substrates of alternate pathways synthesizing steviol glycosides. This could be a plausible reason for maximum reduction in steviol glycoside content on silencing of *SrUGT76G1* than other genes.

**Conclusions:**

*SrKA13H* and *SrUGT85C2* were identified as regulatory genes influencing carbon flux between steviol glycoside and gibberellin biosynthesis. This study has also documented the existence of alternate steviol glycoside biosynthesis route.

## Introduction

*Stevia rebaudiana* is a perennial shrub of Asteraceae family. The scientific research on *S. rebaudiana* and its sweet constituents has increasingly confirmed its nutritional and pharmacological importance [1], [2]. The genus *Stevia* comprises of 220-230 different species. *Stevia rebaudiana* is the only *Stevia* species known so far to contain sweet diterpenoid secondary metabolites, named as steviol glycosides. Steviol glycosides are produced mainly within its leaves. Hence, leaves represent the sweetest part of whole plant [3], [4].

**Figure 1:**
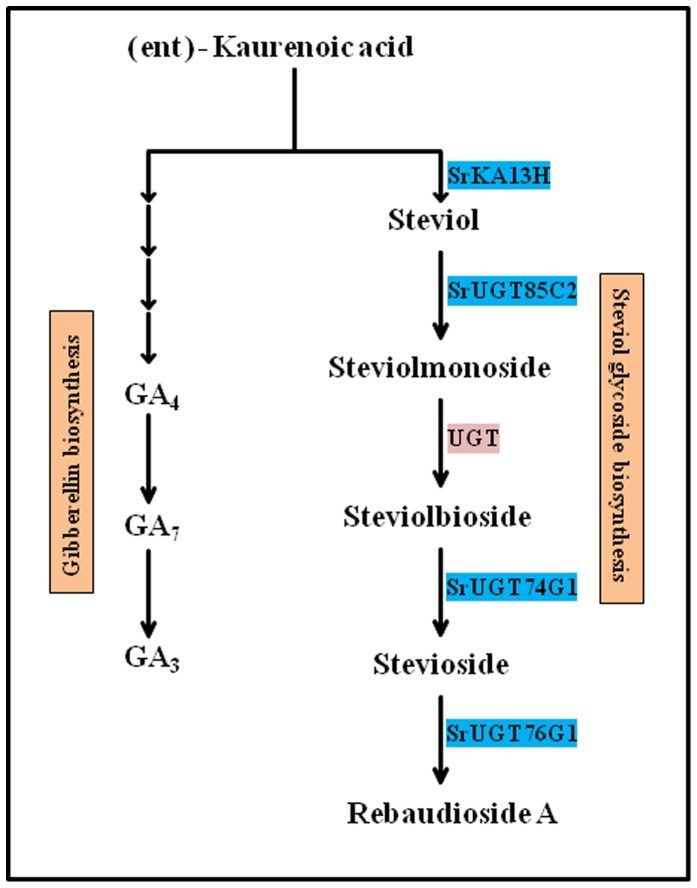
Steviol glycoside biosynthetic pathway. The pathway emerges from the common substrate ent- kaurenoic acid that also gives rise to gibberellin biosynthetic route.

The steviol glycoside biosynthesis pathway synthesizing steviol glycosides comprises of eighteen consecutive enzyme catalyzed steps [5]. Among the eighteen steps, only last five steps have been considered specific to steviol glycoside biosynthesis (Fig. 1). The enzymes of these steps ent-kaurenoic acid-13 hydroxylase and three UDP glycosyltransferases are encoded by genes namely, *SrKA13H*, *SrUGT85C2*, *SrUGT74G1* and *SrUGT76G1*, respectively. One of the enzyme catalyzing an intermediate step is still unknown [5], [6]. Hence, steps catalyzed by enzymes encoded by *SrKA13H*, *SrUGT85C2*, *SrUGT74G1* and *SrUGT76G1* genes have been considered important for steviol glycoside biosynthesis [5]. Steviol glycoside biosynthesis shares common steps with gibberellic acid biosynthesis pathway (Fig. 1). However, the genetic basis of steviol glycoside biosynthesis and its relation with gibberellins is not well understood [6], [7].

In order to understand the genetics of steviol glycoside biosynthesis, functional analysis of pathway specific genes is required. Gene silencing has always been a recommended tool to understand the functionality of genes. RNA interference (RNAi) being the most commonly employed approach, that involves preparation of inverted repeat construct of target gene and generation of transgenics [8]. However, self-incompatibility, genetic heterozygosity and lack of genomic information have been observed to make *Stevia* a difficult system to carry out molecular studies [9]. So, plant breeding has been adopted as a method to study the genetic basis of steviol glycoside production. But this technique involves laborious and time consuming processes of segregation, crossing and selection [3], [9], [10].

Therefore, gene silencing approach was adopted to understand the genetic regulation of steviol glycoside biosynthesis. *Agrobacterium tumefaciens* is being used as an efficient molecular vehicle to transform plants with silencing/overexpression constructs for transgenic development [11]. But *Stevia* lacks a well developed regeneration system [5]. Hence, *Agrobacterium* mediated transient gene silencing (AMTS) could be a better alternative approach. Transient gene silencing has already been identified as proficient alternate for stable transformations. The various reported methods of transient gene silencing involve syringe agroinfiltration [12], vacuum agroinfiltration [13], [14] and virus induced gene silencing [15], [16]. However, vacuum and virus mediated approaches often pose harmful effects on plants [16], [17]. So, in present study AMTS was employed to evaluate the regulation of steviol glycoside biosynthesis and its relation with gibberellin accumulation.

## Materials and Methods

### Plant materials

Seeds of *Stevia rebaudiana* were utilized for raising plants. The seeds were washed twice with autoclaved double distilled water and surface sterilized for ten minutes using 1% (v/v) sodium hypochlorite solution. The seeds were further rinsed thrice with autoclaved double distilled water. These treated seeds were planted on germination medium and allowed to germinate under 16/8 h light/dark tissue culture conditions. The germination media was constituted by standard Murashige and Skoog (MS) salts supplemented with MS vitamins (1000X), 3% (w/v) sucrose and 0.7% (w/v) agar. The pH of media was maintained at 5.85 with 1N sodium hydroxide. The plants were maintained under tissue culture conditions by performing routine sub-culturing of plantlets. Two-three months old tissue culture maintained plants were then transferred to earthen pots and shifted to green house for hardening. Leaves of green house maintained plants were used for RNA isolation and conducting silencing studies.

### Preparation of RNA interference (RNAi) construct

Full length cDNAs encoding ent-kaurenoic acid-13 hydroxylase (*SrKA13H*; Accession no. DQ398871.3) and three UDP glycosyltransferases, *SrUGT85C2* (Accession no. AY345978.1), *SrUGT74G1* (Accession no. AY345982.1) and *SrUGT76G1* (Accession no. AY345974.1) were obtained from Nucleotide database of National Center of Biotechnology Information (NCBI). For RNAi preparation, primers corresponding to partial fragments of size 255, 183, 249 and 189 bp were designed from the cDNA sequences retrieved from NCBI for the genes *SrKA13H*, *SrUGT85C2, SrUGT74G1* and *SrUGT76G1*, respectively (Table 1). The forward and reverse primers were supplemented at 5’ end with the restriction site AscI and SwaI to generate fragments in sense directions, respectively. Similarly to generate fragments for antisense orientation cloning, the restriction sites of SpeI and BamHI were added to forward and reverse primers at 5’ end, respectively. The initially amplified sense and antisense gene specific fragments were individually cloned into pGEMT easy vector. The resulting recombinant pGEMT easy vectors were restriction digested to fish out specific partial (sense-antisense) fragments. The isolated/digested fragments were finally cloned into vector pFGC1008 on either side of GUS intron in an inverted repeat orientation.

**Table 1:** Primer sequences and their annealing conditions.

Gene	Primer sequence	Annealing conditions
SrKA13H	F- CTTCCAATCGACGTCCCAGR-CGAGGTATCATGACCCGC	58°C, 30 sec
SrUGT85C2	F-CCCGCTGGACTGGAGCR-GGGCCGATGGTGTAAATG	56°C, 30 sec
SrUGT74G1	F-GGAATCGATGGTGGTTCGR-GAAGACCCAACGTGCTTG	56°C, 30 sec
SrUGT76G1	F-GGTCCGCTCGCTGGTATGR-CCGTCGGAGGTTAAGACTG	60°C, 30 sec

### *Agrobacterium* mediated transient gene silencing (AMTS)

The prepared silencing constructs were transformed into Agrobacterium tumefaciens strain LBA4404 by triparental mating. The transformed *Agrobacterium* cells were initially grown in 5 ml of liquid YEM supplemented with chloremphenicol and streptomycin at 28°C for 24 h. Two ml of cultured cells were recultured in 10 ml of fresh liquid YEM supplemented with same antibiotics for 36–48 h at 28°C. The culture was pelleted down and resuspended in 5 ml of MMA [1X MS, 10 mM MES (2-(N-morpholino) ethane sulfonic acid), 200 µM acetosyringone, pH 5.6] and recultured for 3 h at 28°C. Afterwards, the culture was again pelleted down and resuspended in 2 ml of 10 mM MgCl_2_ for washing. After washing, pellet was dissolved in 2 ml of 10 mM MgCl_2_ [18]. Syringe agroinfiltration method was used for inoculation of RNAi constructs. The processed culture was taken in a 2 ml syringe and the syringe was used without needle for infiltration to avoid leaf injury. Leaves of six month old green house maintained *Stevia* plants were employed for infiltration. The syringe mouth was placed on the abaxial leaf surface and counterbalanced on the opposite side by thumb or finger. The infiltrated leaf samples were collected on 5, 7, 9, 12 and 14 days post infiltration (dpi). The collected leaf samples were employed for RNA isolation and cDNA synthesis.

### Semi-quantitative PCR analysis

Total RNA was isolated from the infiltrated leaf samples (100 mg) collected at various time points using Qiagen RNeasy Plant Mini kit according to manufacturer’s instructions. First strand cDNA synthesis was conducted using Superscript III RT (Invitrogen) with 1 μg of total RNA and oligodT primers. Equal quantity of synthesized cDNA was employed to evaluate the transcript expression of genes *SrKA13H*, *SrUGT85C2*, *SrUGT74G1* and *SrUGT76G1* using semi-quantitative PCR on AMTS. Gene specific primer sequences and PCR reaction conditions were used as in Table 1. Level of 26S rRNA amplified in each sample with standard primers was employed as internal control [19].

### Quantitation of steviol glycosides

The pre-collected leaf samples were used for total steviol glycoside content estimation as described earlier [20]. Samples (100 mg) were crushed and extracted overnight with 80% methanol at room temperature. Extract was filtered and the filtrate was dried *in vacuo*. The dried extract was defatted with hexane and residual extract was vacuum dried. The resulting extract was dissolved in acetonitrile and filter-sterilized for HPLC analysis. Ten μl of sample was injected to Lichrosphere NH_2_ column using an isocratic solvent system of acetonitrile:water (80:20) at a flow rate of 0.8 ml/min. Steviol glycosides were detected at 205 nm on photodiode array detector. Three independent biological replicates were employed for extraction to ensure reproducibility.

### Estimation of gibberellin (GA_3_)

GA_3_ was estimated from the pre-collected samples of infiltrated *Stevia* leaves following the method described earlier [21]. Sample (100 mg) was crushed with 70% methanol and extracted overnight with continuous shaking at 4°C. The methanolic extract was filtered and vacuum dried. The pH of dried extract was adjusted to 8.5 with 0.1 M phosphate buffer. The obtained solution was partitioned thrice with ethyl acetate and organic phase was discarded. Further, the pH of aqueous phase was adjusted to 2.5 with 1 N HCl. The aqueous phase was then partitioned thrice with diethyl ether. The ether phases were pooled together and evaporated *in vacuo*. The resultant residue was dissolved in acetonitrile and stored at 4°C. Sample (10 µl) was injected into Lichrosphere C_18_ column using the isocratic solvent system of acetonitrile:water (26:74) supplemented with 30 mM phosphoric acid (pH 4.0). Flow rate was maintained at 0.8 ml/min. GA_3_ was detected at 208 nm using photodiode array detector. The gibberellin extraction was performed in three independent biological replicates.

### Bio-informatic prediction of binding affinity of *Stevia* SrUGTs with substrates

Three dimensional structure predicted for proteins encoded by *SrUGT85C2*, *SrUGT74G1* and *SrUGT76G1* [22] were docked to substrates 19-*O*-β-glucopyranosyl steviol and rubusoside. Molecular Docking Server (http://www.dockingserver.com) was employed for the same. The desired substrate molecules were identified from NCBI PubChem and uploaded to docking server along with query protein molecules [23]. Docking run was performed twice to obtain best results. Every single protein molecule was docked with each substrate.

### Statistical analysis

All the measurements were made in triplicate until and unless stated. Data is presented as mean ± standard deviation (SD). Significant differences for various measurements between control and transgenic lines were calculated corresponding to P <0.05 and P <0.01 by Student’s *t*-test.

## Results

### RNA interference (RNAi) construct preparation

**Figure 2:**
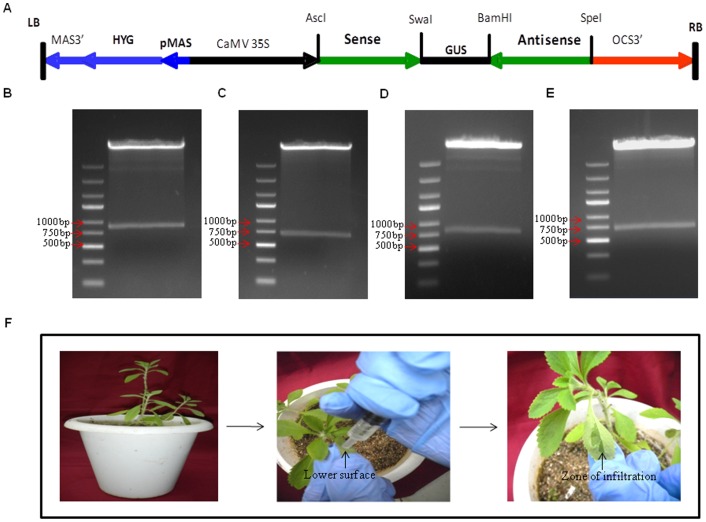
RNA interference (RNAi) construct preparation and agroinfiltration. (A) Diagrammatic representation of RNAi silencing constructs prepared by using pFGC1008 vector backbone. The partial fragments of genes (sense and antisense) were cloned in inverted repeat orientation surrounding the GUS intron. The transgene was under the control of CaMV 35S promoter. Confirmation of silencing constructs preparation by double digestions with restriction enzymes AscI-SpeI. Presence of fragments of size 858, 714, 846 and 726 bp confirmed the preparation of silencing constructs corresponding to the genes (B) *SrKA13H*, (C) *SrUGT85C2*, (D) *SrUGT74G1* and (E) *SrUGT76G1*. (F) Syringe mediated agroinfiltration in *Stevia* leaves.

In order to study the molecular regulation of steviol glycoside biosynthesis pathway, *Agrobacterium* mediated transient gene silencing (AMTS) was conducted. For AMTS, RNAi based silencing constructs were prepared for genes *SrKA13H* encoding ent-kaurenoic acid-13 hydroxylase and *SrUGT85C2*, *SrUGT74G1*, *SrUGT76G1* encoding respective UDP glycosyltransferases. Partial fragments of size 255, 183, 249 and 189 bp analogous to genes *SrKA13H*, *SrUGT85C2*, *SrUGT74G1* and *SrUGT76G1* were cloned in sense and antisense orientation surrounding GUS intron in pFGC1008 vector, respectively (Fig. 2A). The RNAi construct was under the control of constitutively expressing 35S cauliflower mosaic virus promoter. The prepared RNAi constructs were confirmed by double digestion with restriction enzymes AscI and SpeI. Fishing out of fragments of size 858, 714, 846 and 726 bp on double digestion validated the preparation of silencing constructs corresponding to genes *SrKA13H* (Fig. 2B), *SrUGT85C2* (Fig. 2C), *SrUGT74G1* (Fig. 2D) and *SrUGT76G1* (Fig. 2E), respectively. The prepared silencing constructs were finally transformed into *Agrobacterium* strain LBA4404.

### Syringe agroinfiltration of *Stevia* leaves

Among the eighteen known enzymes of this biosynthetic route, enzymes encoded by *SrKA13H*, *SrUGT85C2*, *SrUGT74G1* and *SrUGT76G1* have been considered pathway specific [5], [6]. So, silencing of these four genes would help to understand the mechanism and regulations of this pathway. The prepared RNAi constructs were used to study the regulation of steviol glycoside biosynthesis pathway. Syringe mediated agroinfiltration method was adopted to achieve silencing of these genes. The culture was gently injected into the leaf to prevent leaf damage. The movement of culture was visualized with naked eye. The success of infiltration was estimated by the visible zone of infiltration present on the leaf surface (Fig. 2F).

### AMTS reduced gene expression in *Stevia rebaudiana*

**Figure 3:**
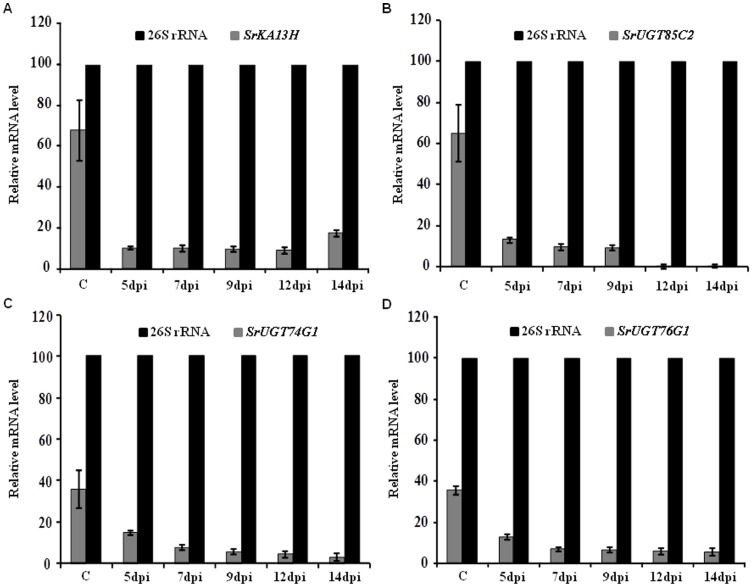
Evaluation of transient gene silencing using semiquantitative-PCR. Transcript expression of steviol glycoside pathway specific genes was analyzed on 5, 7, 9 and 12 days post infiltration (dpi). Untransformed wild was used as control. Transcript expression of 26S rRNA was used as internal control. Bar diagram shows the densitometric quantification of relative mRNA accumulation of (A) *SrKA13H*, (B) *SrUGT85C2*, (C) *SrUGT74G1* and (D) *SrUGT76G1* on agroinfiltration of gene specific RNAi constructs with respect to internal control. The RNAi constructs corresponding to these four genes were found to significantly reduce the transcript expression of respective endogenous genes.

Semi-quantitative PCR analysis was conducted to check the effectiveness of silencing. Leaves infiltrated with RNAi construct of gene *SrKA13H* were observed to decrease mRNA of *SrKA13H* by 74–86% than control in due course of agroinfiltration from 5 to 14 days post infiltration (dpi). The expression of *SrKA13H* transcript was consistently reduced by 86% than control on 5, 7, 9 and 12 dpi (Fig. 3A). In case of *SrUGT85C2* agroinfiltration, 79–99% of relative reduction in transcript expression was observed in due time period of agroinfiltration. The level of mRNA accumulation was reduced by 79% on 5 dpi. The expression was further reduced by 85% on 7 and 9 dpi and by 99% on 12 and 14 dpi (Fig. 3B).

The silencing construct corresponding to gene *SrUGT74G1* was found to decrease mRNA level by 59–92% in comparison to control. A linear fall was observed in mRNA accumulation from 5 dpi to 14 dpi. A decrease of 59% in transcript expression was evident on 5 dpi. Simultaneously, mRNA accumulation was reduced by 78, 84, 87 and 92% than control on 7, 9, 12 and 14 dpi, respectively (Fig. 3C). Likewise, silencing construct prepared for the fragment corresponding to *SrUGT76G1* was found to downregulate the mRNA of *SrUGT76G1* in *Stevia* leaf by 63–84% in contrast to control upon agroinfiltration. A relative decrease of 63% in mRNA level was noticed on 5 dpi. The transcript expression was further decreased by 80% on 7 and 9 dpi. Subsequently, the mRNA level was reduced by 82 and 84% on 12 and 14 dpi, respectively (Fig. 3D). Hence, the RNAi constructs, optimized method of *Agrobacterium* culture and syringe agroinfiltration was able to downregulate the expression of targeted endogenous genes with higher efficiency.

### Decrease in steviol glycoside content of *Stevia* leaves- a consequence of AMTS

**Figure 4:**
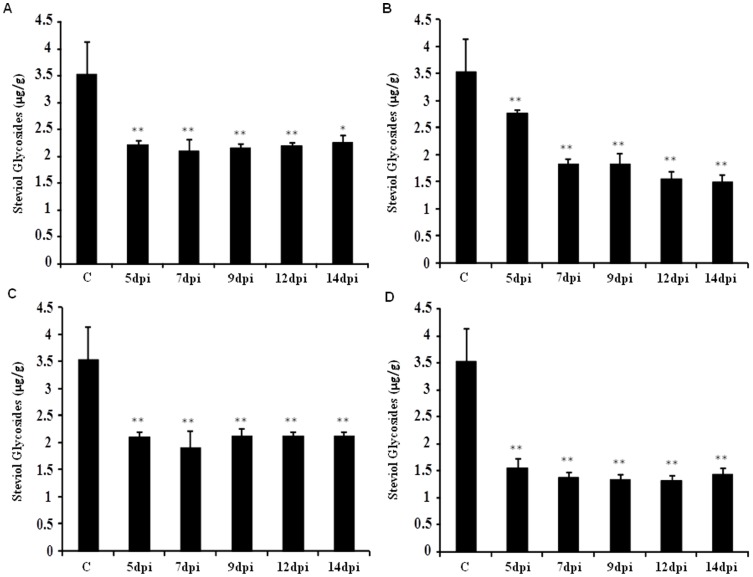
Steviol glycoside content measured on 5, 7, 9 and 12 days post infiltration (dpi) with respect to control. The level of steviol glycosides was estimated using HPLC in leaves of control and agroinfiltrated *Stevia*. Bar diagram shows the steviol glycoside content on AMTS of (A) *SrKA13H*, (B) *SrUGT85C2*, (C) *SrUGT74G1* and (D) *SrUGT76G1*. Data presented as mean±standard deviation of three independent measurements, (**, P<0.01; *P<0.05).

To see the influence of reduced expression of steviol glycoside biosynthesis specific genes on total accumulation of steviol glycosides, leaf samples infiltrated with silencing constructs corresponding to genes *SrKA13H*, *SrUGT85C2*, *SrUGT74G1* and *SrUGT76G1* were analyzed for the estimation of steviol glycosides. Total steviol glycoside content was significantly decreased by 36–40% on transient silencing of gene *SrKA13H* in *Stevia* leaves. The content was maximally decreased by 40% on 7 dpi (Fig. 4A). However, 22–58% decrease of steviol glycoside content was observed on *SrUGT85C2* silencing. The content reduction was 22% on 5 dpi and further dropped by 48% on 7 and 9 dpi. Furthermore, a significant decrease of 56 and 58% in steviol glycoside content was noticed on 12 and 14 dpi, respectively (Fig. 4B).

Downregulation of *SrUGT74G1* transcript expression was found to reduce the level of steviol glycosides by 40–46% at various time points of sampling. The content was reduced maximally by 46% in comparison to control on 7 dpi (Fig. 4C). The transient silencing of *SrUGT76G1* was observed to induce 56–63% decrease in steviol glycoside content. The accumulation of steviol glycosides was significantly reduced from 56 to 61% on 5 to 7 dpi, respectively. The content was further decreased by 62 and 63% on 9 and 12 dpi, respectively (Fig. 4D). Overall, AMTS of *SrUGT76G1* was found to maximally reduce the steviol glycoside content followed by *SrUGT85C2*, *SrUGT74G1* and *SrKA13H*, respectively.

### AMTS induced elevation in GA_3_ content

Steviol glycoside biosynthesis has been reported as a divergence from gibberellic acid biosynthetic route (Fig. 1). As a result, silencing of steviol glycoside biosynthesis genes was expected to alter the accumulation of gibberellic acid. So, the leaf samples infiltrated with respective silencing constructs were employed for GA_3_ estimation using reverse phase high performance liquid chromatography. The limit of detection and limit of quantification of the method was estimated to be 2.26 ng and 7.54 ng of GA_3_, respectively.

**Figure 5:**
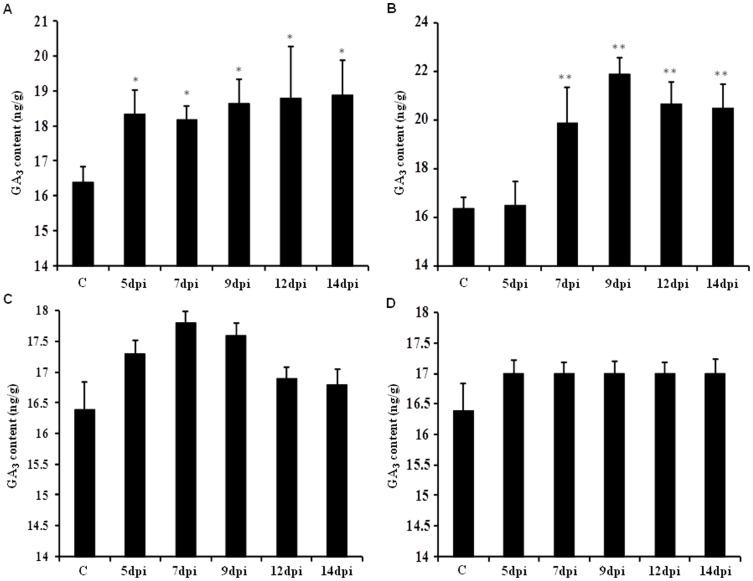
GA_3_ content estimated on 5, 7, 9 and 12 days post infiltration (dpi) with respect to control. Graphical representation of gibberellin content on transient silencing of (A) *SrKA13H*, (B) *SrUGT85C2*, (C) *SrUGT74G1* and (D) *SrUGT76G1*. GA_3_ content was increased to a higher extent on transient silencing of *SrKA13H* and *SrUGT85C2* than noticed during *SrUGT74G1* and *SrUGT76G1* silencing. Data represented as mean±standard deviation of three independent measurements, (**, P<0.01; *, P<0.05).

A significant increase was observed in GA_3_ accumulation on AMTS of *SrUGT85C2* and *SrKA13H*. While, comparatively lesser increase in GA_3_ content was noticed on silencing of *SrUGT74G1* and *SrUGT76G1* transcript. Reduction in *SrKA13H* transcript expression was found to increase the gibberellin level by 11–15% than control. An increment of 12, 11, 14, 15 and 15% in GA_3_ content was noticed in leaves collected on 5, 7, 9, 12 and 14 dpi, respectively (Fig. 5A). AMTS of *SrUGT85C2* was found to increase GA_3_ content by 21–34% than control. Leaf samples collected on 5 dpi of *SrUGT85C2* silencing construct infiltration showed least enhancement in the content than control. However, GA_3_ accumulation was increased by 21 and 34% on 7 and 9 dpi, respectively. Afterwards, the increment was enhanced by 26 and 25% with respect to control on 12 and 14 dpi, respectively (Fig. 5B).

AMTS of *SrUGT74G1* was observed to increase the accumulation of GA_3_ content by 8 and 7% on 7 and 9 dpi, respectively. But subsequently, least change in GA_3_ content was noticed (Fig. 5C). Similarly, transient silencing of *SrUGT76G1* also showed very less increase in gibberellin accumulation. A constant increment of only 4% was noticed on 5 dpi till 14 dpi (Fig. 5D). Overall, GA_3_ content was increased to a greater extent on AMTS of *SrUGT85C2* and *SrKA13H* than noticed during *SrUGT74G1* and *SrUGT76G1* silencing.

### SrUGT76G1 showed highest affinity for the substrates

**Figure 6:**
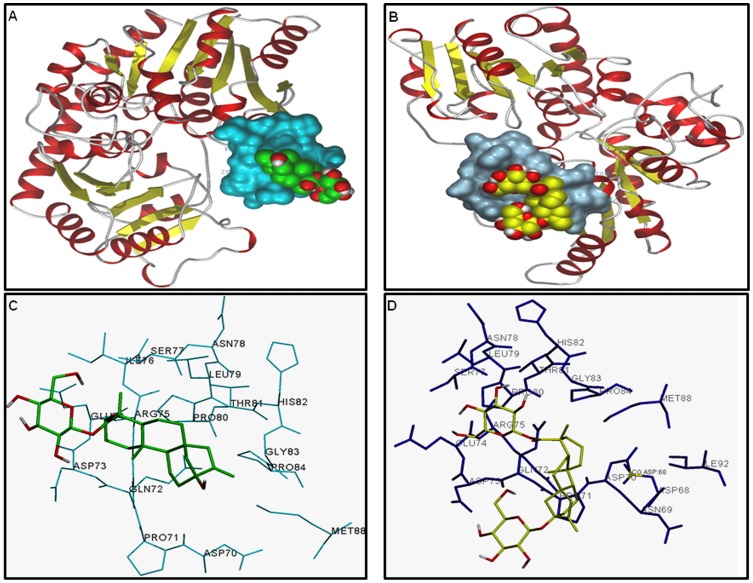
Pictorial representation of SrUGT76G1 protein binding with two substrates of minor pathway, (A) 19-*O*-β-glucopyranosyl steviol and (B) rubusoside. Polar and hydrophobic interactions were involved in protein-ligand binding. (C) A close representation of SrUGT76G1-(19-*O*-β-glucopyranosyl steviol) binding represents the involvement of glutamine, aspartate, glutamate, leucine and proline in the interaction. (D) A close representation of SrUGT76G1-(rubusoside) binding shows the presence of aspartate, proline, glutamine, asparagine, glutamate, leucine and methionine at interacting site.

**Figure 7:**
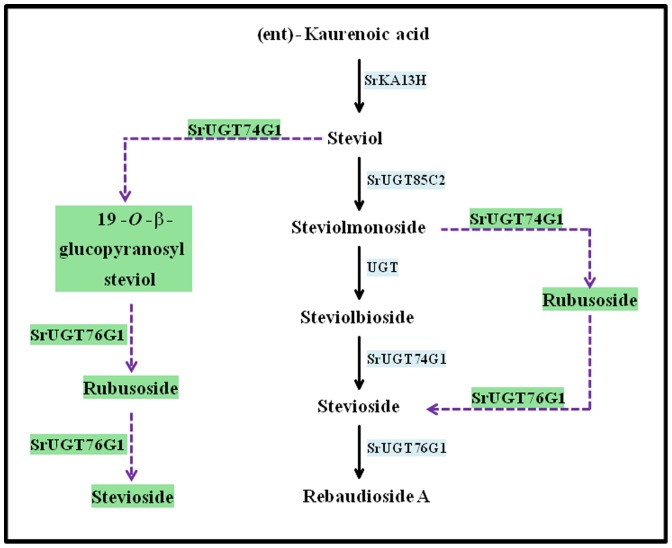
Proposed minor pathways in steviol glycoside biosynthesis. Dotted blue lines and green text boxes indicate the alternate steps and enzymes and substrates engaged in the synthesis of stevioside and rebaudioside A. Present study proposes the catalytic activity of enzyme SrUGT76G1 on substrates 19-O-β-glucpyranosyl steviol and rubusoside, suggesting its involvement in alternate steviol glycoside biosynthesis.

AMTS of *SrUGT76G1* was found to maximally decrease total steviol glycoside content. However, enzyme encoded by *SrUGT76G1* catalyzes only the conversion of stevioside into rebaudioside A (Fig. 1). Since *UGTs* have been reported to act on multiple substrates, three known SrUGTs of steviol glycoside route might be acting in the similar way [24]. So, docking studies were performed to evaluate the affinity of three SrUGT proteins with the substrates 19-*O*-β-glucopyranosyl steviol and rubusoside. As shown in Table 2, the protein SrUGT76G1 showed least binding energy for both the substrates. Least binding energy of the protein model for a substrate documented for its highest binding affinity with the same compared to others [22]. Hence, the geometry for binding of SrUGT76G1 was predicted with 19-*O*-β-glucopyranosyl steviol (Fig. 6A) and rubusoside (Fig. 6B). The protein model predicted for SrUGT76G1 showed polar and hydrophobic interactions of amino acid at positions 72, 73, 74, 79 and 80 with the ligand 19-*O*-β-glucopyranosyl steviol (Fig. 6C). Similarly, amino acids at position 70, 71, 72, 73, 74, 79, 80 and 88 were involved in polar and hydrophobic interactions of SrUGT76G1 with rubusoside (Fig. 6D). The amino acids involved in interaction with 19-*O*-β-glucopyranosyl steviol and rubusoside were also identified. Amino acids glutamine, aspartate, glutamate, leucine and proline were present at the site of interaction with 19-*O*-β-glucopyranosyl steviol (Fig. 6C). Likewise, amino acids aspartate, proline, glutamine, asparagine, glutamate, leucine and methionine at the binding site of SrUGT76G1 were involved in interaction with rubusoside (Fig. 6D). SrUGT76G1 protein model showed higher number of polar and hydrophobic interactions for both the substrates than proteins SrUGT85C2 (Fig. S1) or SrUGT74G1 (Fig. S2). Further, presence of large number of hydrophobic interactions has been known to increase the binding affinity of protein and ligand [25]. So, SrUGT76G1 might be catalyzing 19-*O*-β-glucopyranosyl steviol and rubusoside to synthesize rubusoside and stevioside, respectively (Fig. 7). Thus, enzyme encoded by *SrUGT76G1* might be involved in alternate biosynthesis of steviol glycosides in addition to its catalytic role in the main biosynthetic route (Fig. 7).

**Table 2:** Binding energy (kCal/mol) of SrUGTs of steviol glycoside biosynthesis pathway with the substrates 19-*O*-β-glucopyranosyl steviol and rubusoside.

Protein/Substrate	19-*O*-β-glucopyranosyl steviol	Rubusoside
SrUGT85C2	–4.42	+4.08
SrUGT74G1	–5.15	+16.36
SrUGT76G1	–7.11	–4.79

Lowest the binding energy, highest will be the binding affinity.

## Discussion

Steviol glycoside biosynthesis in *Stevia* is peculiar in a way that it emerges as a deviation of gibberellin biosynthesis [6], [7]. Further to bifurcation, the steps catalyzed by enzymes ent-kaurenoic acid-13 hydroxylase and three UDP-glycosyltransferases encoded by genes *SrKA13H*, *SrUGT85C2*, *SrUGT74G1* and *SrUGT76G1*, respectively are involved in the synthesis of steviol glycosides [5]. However due to lack of genomic information of *Stevia*, the detailed mechanistic and regulatory studies of steviol glycoside biosynthesis are lacking. Hence with the aim of studying the molecular mechanism and regulation of steviol glycoside biosynthesis, *Agrobacterium* mediated transient gene silencing (AMTS) of *SrKA13H*, *SrUGT85C2*, *SrUGT74G1* and *SrUGT76G1* was conducted in present study. The gene *SrKA13H* designated by accession number DQ398871.3 has been employed in the present study to conduct AMTS, since the same gene has been documented to successfully synthesize steviol in microbial systems [26]. Further, another independent study has documented the role of this gene in steviol production in prokaryotic expression system [27].

Among the various known methods of transient gene silencing, syringe agroinfiltration has been considered suitable [12], [13], [14], [15], [16]. Most recently, virus induced gene silencing via agroinfiltration has been reported for *Gerbera hybrida*, another member of Asteraceae family. It has been documented that vacuum infiltration of *Agrobacterium* transformed VIGS vector offered intensive silencing. However, simultaneous occurrence of mechanical plant injury due to vacuum and disease symptoms due to virus infection were also reported in the transformed *Gerbera* plants [16]. Hence in present study, syringe agroinfiltration method was successfully adopted to conduct transient silencing in *Stevia rebaudiana*.

Infiltration of silencing constructs corresponding to genes *SrKA13H* and three *SrUGTs* was found to significantly downregulate the expression of respective endogenous genes. The variability obtained in reduction of *SrKA13H* and *SrUGTs* transcript expression could be due to their variable mRNA accumulation under control conditions [28]. Semi-quantitative PCR analysis has shown that mRNA accumulation of *SrKA13H* and *SrUGT85C2* was higher than *SrUGT74G1* and *SrUGT76G1* in *Stevia* leaves. The expression of *SrUGT85C2* has also been noticed higher than rest of the two *SrUGTs* [29]. Hence, higher mRNA level of *SrKA13H* and *SrUGT85C2* might have facilitated higher degradation of their mRNAs on AMTS than *SrUGT74G1* or *SrUGT76G1*. Likewise in *soybean*, the VIGS of *CHS* gene has been reported to downregulate the transcript expression more in seed coat than in leaves. Higher accumulation of *CHS* mRNA in seed coat than leaves has been suggested for such differential VIGS silencing effects [28].

*SrKA13H*, *SrUGT85C2*, *SrUGT74G1* and *SrUGT76G1* genes have been considered important for steviol glycoside biosynthesis. Consequently, their downregulation was found to negate the accumulation level of steviol glycosides. However, reduction in total steviol glycoside content was not more than 60% on AMTS of any of the four genes. Hence, alternate pathways might be present that could synthesize steviol glycosides on silencing of any of the genes of major pathway. Similarly, downregulation of caffeine synthase (*CS*) gene in *Camellia sinensis* has also hinted for the presence of minor pathways for the synthesis of caffeine in addition to its known major biosynthetic pathway [30].

*UGTs* have been considered multi-substrate acting enzymes that are regio-specific and regio-selective [24]. *SrUGTs* (*SrUGT85C2*, *SrUGT74G1* and *SrUGT76G1*) of steviol glycoside biosynthesis have earlier been analyzed for their reactivity against various known substrates of the pathway [31], [32]. The *in vitro* analysis has led to the introduction of various bifurcations to main biosynthetic route. SrUGT74G1 has been predicted to catalyze steviol into 19-*O*-β-glucopyranosyl steviol (Fig. 7). This compound subsequently gives rise to rubusoside and stevioside. But enzymes catalyzing these conversions have not been identified. Further, SrUGT74G1 has also been predicted to synthesize rubusoside by acting on steviolmonoside (Fig. 7). Rubusoside is again metabolized to stevioside and the acting enzyme is still unknown [31]. Additionally, *in vivo* existence of these bifurcations have not been reported or known [31]. In this respect, present study documented highest affinity of protein SrUGT76G1 for 19-*O*-β-glucopyranosyl steviol and rubusoside. These metabolites have earlier been predicted as intermediate substrates of alternate pathways synthesizing steviol glycosides. However, their position in the pathway was not decided [31], [32]. Thus, enzyme encoded by *SrUGT76G1* might be involved in alternate routes of steviol glycoside biosynthesis by catalyzing 19-*O*-β-glucopyranosyl steviol and rubusoside, in addition to the catalysis of stevioside into rebaudioside A. So, silencing of *SrUGT76G1* could have blocked these minor pathways in addition to the main biosynthetic route. This could be possible reason for the reduction in steviol glycoside content to a greater extent upon *SrUGT76G1* silencing compared to the silencing of other genes.

Earlier, *in vitro* analysis and HPLC assays have documented equal affinity of SrUGT74G1 for steviolmonoside as well as steviolbioside. SrUGT74G1 acts upon steviolmonoside to synthesize rubusoside. While stevioside was synthesized from steviolbioside by the catalytic action of SrUGT74G1 (Fig. 6). It has been documented that SrUGT74G1 preferably synthesized rubusoside by catalyzing steviolmonoside, if equal preference for both steviolmonoside and steviolbioside was given. Earlier, rubusoside was not reported in *Stevia* plant. Hence, steviolbioside was considered as an intermediate and not the rubusoside [31]. But isolation of rubusoside from *Stevia* has now been reported that suggests the possible involvement of this compound in steviol glycoside biosynthesis [33]. Additionally, rubusoside has been considered superior to stevioside because latter possessed a bitter after-taste [34]. Thus, it again suggests the involvement of minor pathways in steviol glycoside biosynthesis, probably in a region specific or selective manner. Overall, highest affinity of enzyme SrUGT76G1 for substrates 19-*O*-β-glucopyranosyl steviol and rubusoside and maximum reduction of steviol glycoside content on AMTS of *SrUGT76G1* documents the *in vivo* occurrence/involvement of 19-*O*-β-glucopyranosyl steviol and rubusoside in synthesis of steviol glycosides.

Steviol glycosides and gibberellins have common route of origin [6], [7]. These biosynthetic routes bifurcate as two different pathways after the common metabolite ent-kaurenoic acid (Fig. 1). Earlier, various studies have documented the reliability of HPLC based UV detection of gibberellin, GA_3_ from several plant species. Further, as HPLC based method does not require derivatization of gibberellins, it has been documented as a simple method [21], [35], [36], [37]. So due to the ease of optimization, simplicity and rapidity, HPLC based UV-Vis spectroscopy detection was employed for quantification of GA_3_ in present study. The downregulation of steviol glycoside pathway specific genes was found to induce increment in the content of bioactive gibberellin, GA_3_. However, GA_3_ content was increased comparatively to a greater extent on AMTS of *SrKA13H* and *SrUGT85C2* than *SrUGT74G1* or *SrUGT76G1*. Transient silencing of *SrKA13H* might have left ent-kaurenoic acid free to be converted into gibberellins, hence increasing the same. Whereas steviol might have been left unreacted on silencing of *SrUGT85C2*. Earlier, it has been documented that steviol could act as a precursor for the synthesis of C-13 hydroxy-gibberellins [38]. So, downregulation of *SrUGT85C2* might have caused the conversion of steviol into C-13 hydroxy-gibberellins, thus increasing the accumulation of bioactive gibberellins GA_3_. In addition, silencing of *SrKA13H* and *SrUGT85C2* has blocked the metabolite flow of steviol glycoside biosynthesis. This might be responsible for diverting the carbon flux towards gibberellin biosynthesis. Blockage of one of the bifurcation chain (steviol glycoside route) of a common metabolite (ent-kaurenoic acid) could have possibly enhanced the metabolic input and reaction rate of the other diverging route (gibberellin route). Hence, *SrKA13H* and *SrUGT85C2* were found to channelize metabolic flux between steviol glycoside and gibberellin biosynthetic pathways. However, gibberellin content was least affected on AMTS of *SrUGT74G1* and *SrUGT76G1*. This could be because enzymes encoded by these genes catalyze the downstream steps of steviol glycoside biosynthesis. Further, *SrUGT74G1* and *SrUGT76G1* may not be the regulatory genes of steviol glycoside pathway.

In conclusion, *SrKA13H* and *SrUGT85C2* are two important genes encoding regulatory enzymes of steviol glycoside biosynthetic route. Data also suggests the presence of alternate steps in steviol glycoside biosynthesis. Our study documents for the first time *in vivo* evidence suggesting involvement of 19-*O*-β-glucopyranosyl steviol and rubusoside in steviol glycoside biosynthesis. Further, the presence of alternate/minor steps could be spatially and temporally regulated.

## Supporting Information

Figure S1 Pictorial representation of SrUGT85C2 protein binding with two substrates of minor pathway, (A) 19-*O*-β-glucopyranosyl steviol and (B) rubusoside. Polar and hydrophobic interactions were involved in protein-ligand binding. (C) A close representation of SrUGT85C2-(19-*O*-β-glucopyranosyl steviol) binding represents the involvement of aspartic acid, asparagines and phenylalanine in the interaction. (D) A close representation of SrUGT85C2-(rubusoside) binding shows the presence of asparagine and phenylalanine at interacting site.
(JPG)

Figure S2 Pictorial representation of SrUGT74G1 protein binding with two substrates of minor pathway, (A) 19-*O*-β-glucopyranosyl steviol and (B) rubusoside. (C) A close representation of SrUGT74G1-(19-*O*-β-glucopyranosyl steviol) binding represents the involvement of glutamic acid in polar interaction. (D) A close representation of SrUGT74G1-(rubusoside) binding shows the presence of glutamic acid and methionine at interacting site. Polar and hydrophobic interactions were involved in protein-ligand binding.
(JPG)
