# Supplementary figures and images for: Agrobacterium Mediated Transient Gene Silencing (AMTS) in Stevia rebaudiana: Insights into Steviol Glycoside Biosynthesis Pathway
Source: PLoS One. 2013 Sep 4;8(9):e74731. doi: 10.1371/journal.pone.0074731 (PMC3762721; doi:10.1371/journal.pone.0074731)

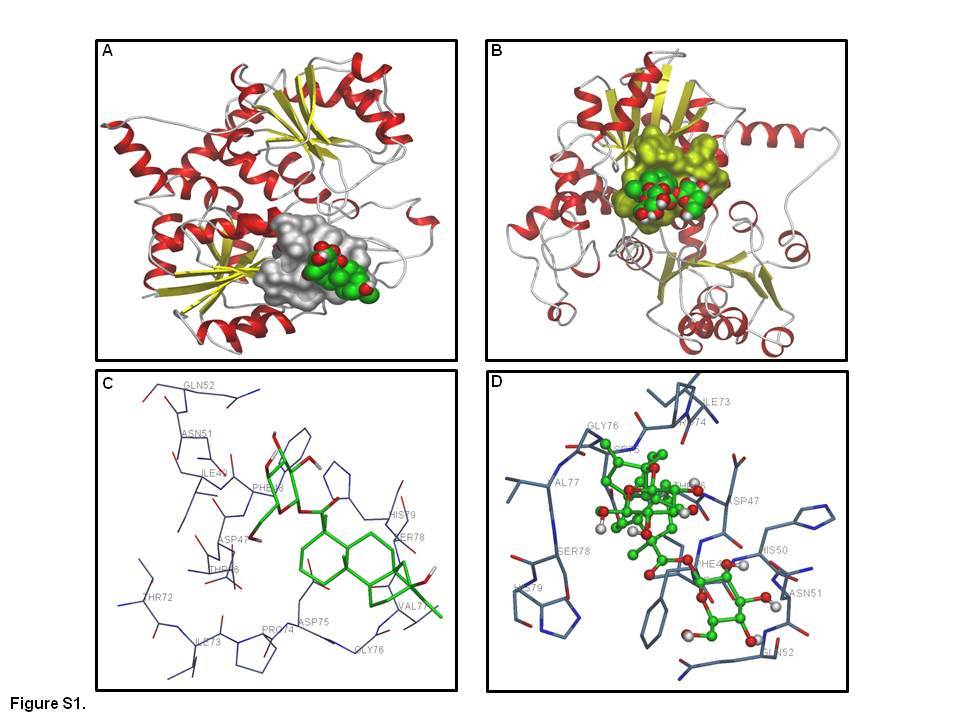

Supplement: Figure S1 — Pictorial representation of SrUGT85C2 protein binding with two substrates of minor pathway, (A) 19-O-β-glucopyranosyl steviol and (B) rubusoside. Polar and hydrophobic interactions were involved in protein-ligand binding. (C) A close representation of SrUGT85C2-(19-O-β-glucopyranosyl steviol) binding represents the involvement of aspartic acid, asparagines and phenylalanine in the interaction. (D) A close representation of SrUGT85C2-(rubusoside) binding shows the presence of asparagine and phenylalanine at interacting site. (JPG) [file pone.0074731.s001.jpg]

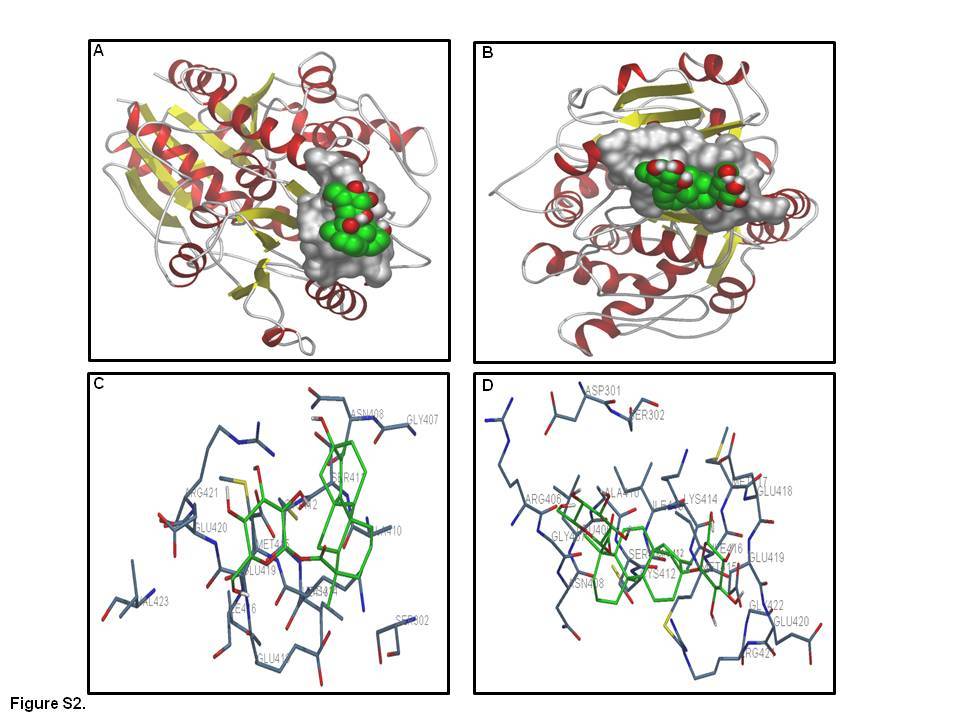

Supplement: Figure S2 — Pictorial representation of SrUGT74G1 protein binding with two substrates of minor pathway, (A) 19-O-β-glucopyranosyl steviol and (B) rubusoside. (C) A close representation of SrUGT74G1-(19-O-β-glucopyranosyl steviol) binding represents the involvement of glutamic acid in polar interaction. (D) A close representation of SrUGT74G1-(rubusoside) binding shows the presence of glutamic acid and methionine at interacting site. Polar and hydrophobic interactions were involved in protein-ligand binding. (JPG) [file pone.0074731.s002.jpg]
